# Insights gained from the reverse engineering of gene networks in keloid fibroblasts

**DOI:** 10.1186/1742-4682-8-13

**Published:** 2011-05-02

**Authors:** Brandon NS Ooi, Toan Thang Phan

**Affiliations:** 1 Graduate Programme in Bioengineering, National University of Singapore, Singapore; 2 Department of Surgery, National University of Singapore, Singapore

## Abstract

**Background:**

Keloids are protrusive claw-like scars that have a propensity to recur even after surgery, and its molecular etiology remains elusive. The goal of reverse engineering is to infer gene networks from observational data, thus providing insight into the inner workings of a cell. However, most attempts at modeling biological networks have been done using simulated data. This study aims to highlight some of the issues involved in working with experimental data, and at the same time gain some insights into the transcriptional regulatory mechanism present in keloid fibroblasts.

**Methods:**

Microarray data from our previous study was combined with microarray data obtained from the literature as well as new microarray data generated by our group. For the physical approach, we used the fREDUCE algorithm for correlating expression values to binding motifs. For the influence approach, we compared the Bayesian algorithm BANJO with the information theoretic method ARACNE in terms of performance in recovering known influence networks obtained from the KEGG database. In addition, we also compared the performance of different normalization methods as well as different types of gene networks.

**Results:**

Using the physical approach, we found consensus sequences that were active in the keloid condition, as well as some sequences that were responsive to steroids, a commonly used treatment for keloids. From the influence approach, we found that BANJO was better at recovering the gene networks compared to ARACNE and that transcriptional networks were better suited for network recovery compared to cytokine-receptor interaction networks and intracellular signaling networks. We also found that the NFKB transcriptional network that was inferred from normal fibroblast data was more accurate compared to that inferred from keloid data, suggesting a more robust network in the keloid condition.

**Conclusions:**

Consensus sequences that were found from this study are possible transcription factor binding sites and could be explored for developing future keloid treatments or for improving the efficacy of current steroid treatments. We also found that the combination of the Bayesian algorithm, RMA normalization and transcriptional networks gave the best reconstruction results and this could serve as a guide for future influence approaches dealing with experimental data.

## Background

Keloids are large protruding claw-like scars that extend well beyond the confines of the original wound and do not subside with time [1]. They uniquely affect only humans, and may develop even after the most minor of skin wounds, such as insect bites or acne [2]. Keloids are frequently associated with itchiness, pain and, when involving the skin overlying a joint, restricted range of motion [3]. It is not well documented how commonly keloids occur in the general population but the reported incidence range from a high of 16% among adults in Zaire to a low of less than 1% among adults in England [4]. In a study assessing the quality of life of patients with keloid and hypertrophic scarring, it was demonstrated for the first time that the quality of life of these patients was reduced due to physical and/or psychological effects [5]. The problem is further exacerbated by the fact that there is no particularly effective treatment to date [6,7]. Keloids also have a propensity to recur after surgery and have been considered as benign tumours [4].

The goal of reverse engineering methods is to infer gene networks from observational data, thus providing insight into the inner workings of a cell [8,9]. There are two general strategies for reverse engineering gene networks - a physical approach where physical interactions between transcription factors (TFs) and their promoters are modeled, and an influence approach where the mechanistic process is abstracted out as a black box [10]. The advantage of the physical approach is that it enables the use of genome sequence data, in combination with RNA expression data, to enhance the sensitivity and specificity of predicted interactions, but its limitation is that it cannot describe regulatory control by mechanisms other than transcription factors. On the other hand, an advantage of the influence strategy is that the model can implicitly capture regulatory mechanisms at the protein and metabolite level that are not physically measured, but the limitation is that it can be difficult to interpret in terms of the physical structure of the cell. Moreover, the implicit description of hidden regulatory factors may lead to prediction errors [10].

In addition to these two modeling approaches, reverse engineering methods also differ in terms of the mathematical formalisms used and can be static or dynamic, continuous or discrete, linear or nonlinear and deterministic or stochastic [11]. For the purposes of this study, we have chosen to use both the physical as well as the influence approach for reconstructing the networks. For the physical approach, we will use the regression method fREDUCE (fast-Regulatory Element Detection Using Correlation with Expression) [12] with the objective of identifying important cis-binding motifs and their targets in keloid fibroblasts. For the influence approach, we will compare the performance of the information theoretic method ARACNE (Algorithm for the Reconstruction of Accurate Cellular Networks) [13] and the Bayesian package BANJO (Bayesian Network Inference with Java Objects) [14] in uncovering regulatory interactions in keloid and normal fibroblasts. The effect of different normalization/summarization methods and lowly expressed probes on gene network inference will also be examined in this system.

Microarray data from previous studies will be used to learn the networks. However, learning the structure of a gene network using the influence approach is difficult as the number of possibilities scale exponentially with the number of variables. Therefore, modeling and testing such large structures would require large amounts of data for accuracy. Due to our limited data, we have decided to focus on small networks of genes that have been found to be differentially expressed from our previous work. Furthermore, to increase the number of samples, we will also use data from Smith et al [15], which is the only keloid fibroblast data publicly available at the Gene Expression Omnibus (GEO) database. For the physical approach, since the binding motif repeats are regressed against the expression levels of each gene, it is the number of genes that constitute the sample size. Therefore, the full range of genes is used for this approach instead of the smaller transcriptional networks that have found to be differentially expressed.

In total, we have four different treatment conditions (serum-treated, serum-free, hydrocortisone-treated and HDGF-treated) and two different cell derivations (keloid and normal) from multiple patients. Although some of our datasets consist of time-series data, the gap between each time point is very large (in the order of days) and may lead to inaccurate results if used to infer time-series regulatory networks. Therefore, we have limited our study to steady state conditions with the assumption that each time point is statistically independent from others. This is a possibly valid assumption as the sampling time is very long. Furthermore, the genes were not directly perturbed by knockdown or overexpression in our experiments and it is very likely that the different conditions used will result in multiple unknown perturbations. As such, inference algorithms such as dynamic Bayesian networks (which require numerous closely spaced time points) and differential equation approaches (which require either time series data or knowledge of perturbations) cannot be applied in our case.

To date, most attempts at modeling biological networks have been done using simulated data. We hope that this work would highlight some of the issues involved in working with experimental data. Furthermore, we also hope that insights gained from this endeavor would provide some clues about the different transcriptional regulatory mechanisms present in keloid and normal fibroblasts.

## Methods

### Keloid and normal fibroblast database

Keloid and normal fibroblasts were selected from a specimen bank of fibroblast strains derived from excised keloid specimens. All patients had received no previous treatment for the keloids before surgical excision. A full history was taken and an examination performed, complete with color slide photographic documentation before taking informed consent prior to excision. Approval by the NUS Institutional Review Board, NUS-IRB was sought before excision of human tissue and collection of cells. Remnant dermis from keloid or normal skin was minced and incubated in a solution of collagenase type I (0.5 mg/ml) and trypsin (0.2 mg/ml) for 6 h at 37°C. The cells were pelleted and grown in tissue culture flasks. The cell strains were maintained and stored in liquid nitrogen until use.

### Cell culture

Five different keloid fibroblast samples and five different normal fibroblast samples that were previously maintained and stored at -150°C were thawed and used for the experiments. Fibroblasts were seeded in 15 cm dishes at a density of 1 × 10^4 ^cells/ml in 10% FCS until confluency and subsequently starved in a serum-free medium for 48 hrs. After 48 hrs, the serum free medium was replaced and fibroblasts were harvested after another 24 hrs (day 1), 72 hrs (day 3) and 120 hrs (day 5). Cells were grown and processed in five batches. Each batch consisted of one keloid and one normal sample harvested at the three different time points. KF1, NF1, KF2, NF2, KF4, NF4 and KF5, NF5 were samples from different patients while KF3 and NF3 were samples from the same patient. In another experiment, one keloid fibroblast sample was grown, treated with hepatoma derived growth factor (HDGF) and harvested for RNA after 6 hours, day 1 and day 2.

### RNA extraction, cRNA preparation and labeling

RNA was extracted using the RNeasy-kit (Qiagen, Hilden, Germany) according to the manufacturer's protocol. Purified RNA was quantified by UV absorbance at 260 and 280 nm on a ND1000 spectrophotometer (Nanodrop™, ThermoScientific). Labeled complementary RNA (cRNA) was produced from total RNA using the GeneChip One-Cycle or Two-Cycle Eukaryotic Target Labeling and Control Reagents (Affymetrix, Santa Clara, USA) according to the manufacturer's protocol.

### Affymetrix chip hybridization and scanning

Fragmented cRNA was then hybridized to preequilibrated Affymetrix GeneChip U133A or the newer Genechip U133 2.0 Plus arrays at 45 °C for 15 hours. The cocktails were removed after hybridization and the chips were washed and stained using Affymetrix wash buffers and stain cocktails in an automated fluidic station. The chips were then scanned in a Hewlett-Packard ChipScanner (Affymetrix, Santa Clara, USA) to detect hybridization signals.

### Data preprocessing

In addition to microarray data generated by our lab, raw microarray data in the form of .CELS files from Smith et al's experiments were also downloaded from the GEO database [15]. Following data collection, RMA and MAS 5.0 normalization and summarization were done using the R Bioconductor package. The four different datasets (serum starvation dataset using U133A arrays, serum starvation dataset using U133 Plus 2.0 arrays, HDGF dataset using U133 Plus 2.0 arrays and Smith's dataset using U133 Plus 2.0 arrays) were normalized and summarized independently. Two different custom Chip Definition Files (CDF) were used [16]. The first CDF was based on the Ensembl Gene database for analysis with fREDUCE as it is easy to obtain the upstream sequence which is required by fREDUCE from the Ensembl database. The second was based on the Entrez Gene database for influence based reverse engineering methods such as BANJO and ARACNE as these probe mappings allow one to ignore any differential signal due to multiple probesets and gives a single value for a given gene. In addition, two lists were produced. In the first list, no filtering was done while in the second list, 25% of the lowly expressed genes were filtered.

### Application of the fREDUCE algorithm

Human genomic sequences 1000 base pairs upstream from the transcriptional start site if known, or from the initiation codon, were extracted from the Ensembl database [17]. As fREDUCE requires only a single expression dataset and makes use of the entire genomic dataset (both signal and background), the datasets were compared as follows: A: Keloid versus normal fibroblasts under serum starvation conditions (only KF1, KF2, NF1 and NF2 were used to keep the number of samples close to the other conditions), B: Keloid versus normal fibroblasts under serum conditions (from Smith et al's dataset), C: Keloid treated with steroid versus serum induced keloid fibroblasts (from Smith et al's dataset), D: Normal treated with steroid versus serum induced normal fibroblasts (from Smith et al's dataset), E: Keloid versus normal fibroblasts both treated with steroid (from Smith et al's dataset) and F: Keloid treated with HDGF versus untreated keloid fibroblasts (from HDGF dataset). The expression value for each gene is represented as the following t-statistic:

where g is the index over genes, μ_e_^g ^is the mean value of gene g under our condition of interest, μ_c_^g ^os the mean value of gene g under control conditions, Var_e_^g ^is the variance of gene g under our condition of interest, Var_c_^g ^is the variance of gene g under control conditions, and n_e _and n_c _are the number of samples under our condition of interest and under control conditions respectively. This statisitic is similar to the z-statistic used by the fREDUCE creators [14]. We then ran fREDUCE on the t-statistic for RMA normalized and MAS 5.0 normalized as well as unfiltered and filtered gene lists on the basis that a higher t-statistic translates to higher expression. Four different sets of parameters were run on each replicate: length 6 with 0 IUPAC substitutions, length 6 with 1 IUPAC substitution, length 7 with 0 IUPAC substitutions and length 7 with 1 IUPAC substitution. Top and consistent binding sequences obtained from fREDUCE above were then searched through the TRANSFAC database [18] for possible gene targets and their corresponding transcription factors. Only gene targets identified from Homo sapiens were collected, and binding sites for all these targets were reconfirmed to be located within the 1000 base pair upstream sequences collected from the Ensemble database previously.

### Pathways selected for influence approach

**Figure 1:**
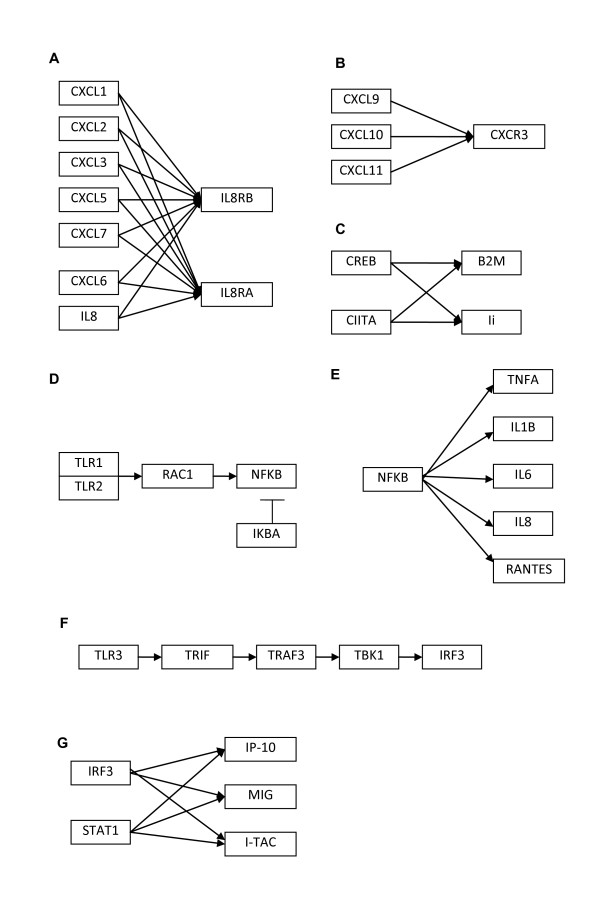
**KEGG pathways used for the influence approach**. (A and B) Pathways taken from the cytokine-cytokine receptor interaction map. (C) Transcriptional pathway taken from antigen processing and presentation map. (D, E, F and G) Pathways taken from the toll-like receptor signaling map.

KEGG pathways that were found to be enriched when comparing keloid to normal fibroblasts from a previous study were used for the influence approach (unpublished data). These were the antigen presentation and processing pathway, cytokine-cytokine receptor interaction and toll-like receptor signaling pathway. Genes that were used as nodes for modeling were chosen on the basis that there is only one gene representing that particular node, all other genes will be assumed to be hidden nodes. The following 5 pathways were eventually selected for the influence approach (Figure 1). Pathways were also chosen such that 1A and 1B represent cytokine receptor interactions, 1C, 1E and 1G represent transcriptional networks and 1D and 1F represent intracellular signaling.

### Application of the ARACNE and BANJO algorithms

Expression values of selected genes from all the different data sets available were used for the influence approach. To enable comparison between the different data sets, gene expression for all the relevant nodes were normalized using the average of GAPDH and B-actin expression. GAPDH and B-actin were first plotted to determine their correlation and outliers were removed from the dataset. Three keloid experiments from the serum starvation U133A dataset did not meet this criteria and was removed giving a total of 28 keloid experiments and 24 normal experiments. We ran ARACNE and BANJO on the keloid and normal inputs separately, and also on the MAS 5 and RMA normalized expression values separately. All parameters were left at their default values. For ARACNE, kernel width and number of bins were automatically detected by the software while DPI tolerance to remove false positives was set at 0.15. For BANJO, the Proposer/Searcher strategies were chosen as random local move and simulated annealing, respectively, and the amount of time BANJO uses to explore the Bayesian Network space was set to one minute. All the other parameters such as reannealingTemperature, coolingFactor, and so on, were left with their default values. Parameter values were selected as best values (in terms of network inference accuracy) as shown by Bansal et al [19]. In order to estimate the joint probability distribution of all variables in the network, BANJO requires discrete data. The data was therefore discretized into 7 discrete states using the quantile discretization procedure in the software. Furthermore, as the simulated annealing algorithm in BANJO does not guarantee a global maximum, the runs were repeated three times and the result with the highest maximum score was taken.

### Estimation of the performance of the algorithms

In order to assess the inference performances we computed the Positive Predicted Value (PPV) and the Sensitivity scores as described by Bansal et al [19]. The following definitions were used:

TP = Number of True Positives = number of edges in the real network that are correctly inferred; FP = Number of False Positives = number of inferred edges that are not in the real network; FN = Number of False Negatives = number of edges in the real network that are not inferred.

The following were then computed:

In order to compute the random PPV we considered the expected value of a hypergeometrically distributed random variable whose distribution function and expected value are, respectively:

where N = number of possible edges in the network, M = number of true edges and n = number of predicted edges. Then,

All statistical tests are done using the one tailed paired t-test.

## Results

### Binding motifs found from fREDUCE for keloid versus normal fibroblasts under serum starvation condition

Binding motifs found using the gene expression values from set A (keloid versus normal fibroblasts under serum starvation conditions) are shown in Table 1. Highlighted motifs indicate top motifs or motifs found in at least two variations of the conditions/parameters. Both MAS5 and RMA normalization as well as filtered and unfiltered gene lists provided hits for the binding motifs. Of particular note are the binding motifs CGCCGA (found in 5 of the conditions), GCCGAC (found in 3 of the conditions), and CACATAT (found in 3 of the conditions). A search through the TRANSFAC database did not produce any results for the binding motif CACATAT, but found possible gene targets for CGCCGA (MYB) and GCCGAC (ATF2) (Table 2).

**Table 1:** Binding motifs found from fREDUCE for keloid versus normal fibroblasts under serum starvation condition (P > 1

Normalization	Parameters	Binding Motif	P-value	Correlation
MAS 5 (unfiltered)	Length 7 (0 IUPAC)	***CCGGCC ***	5.31	0.0558
		***GCCGAC***	1.99	0.0432
	
	Length 7 (1 IUPAC)	***CGCBGA***	5.30	0.0605
		MCGGAA	1.42	0.0469

RMA (unfiltered)	Length 7 (0 IUPAC)	***GCCGAC***	3.35	0.0487
		***CACATAT***	2.56	-0.0480
	
	Length 7 (1 IUPAC)	***GBCGAC***	3.56	0.0549
		***CACATAT***	2.02	-0.0470

MAS 5 (filtered)	Length 7 (0 IUPAC)	***CGCCGA***	2.86	0.0616
	
	Length 7 (1 IUPAC)	***CGCCBA***	3.65	0.0726

RMA (filtered)	Length 7 (0 IUPAC)	***CGCCGA***	2.58	0.0561
		TATACAC	1.95	-0.0560
	
	Length 7 (1 IUPAC)	***CACAKAT***	2.33	-0.0649
		***CGCCGA***	2.03	0.0548

Note: P-values are shown as -log_10 _values.

IUPAC characters M = C/A; Y = C/T; K = T/G; B = C/T/G, V = A/C/G

**Table 2:** Possible gene targets and TFs found from the TRANSFAC database for top binding motifs from Table 1

Binding Motif	Possible gene targets	Possible TFs
***CCGGCC***	MC2R (melanocortin 2 receptor)	SF-1
	MT1G (metallothionein 1G)	-
	EPO (erythropoietin)	Tf-LF1 and Tf-LF2
	SURF1 and SURF2 (surfeit 1 and 2)	YY1

***GCCGAC***	ATF2 (activating transcription factor 2)	SP1

***CGCCGA***	c-myb	MZF-1

***CACATAT***	-	-

### Binding motifs found from fREDUCE for keloid versus normal fibroblasts under serum induced condition

No binding motifs were found for unfiltered RMA normalized set B (keloid versus normal fibroblasts under serum conditions), but binding motifs were found for the other conditions (Table 3). Of particular note is the binding motif GGGGCTC which was found to be consistent in 4 of the conditions, although all these 4 conditions were using the MAS 5 normalization. A search through the TRANSFAC database found ADA as a possible gene with this binding motif (Table 4).

**Table 3:** Binding motifs found from fREDUCE for keloid versus normal fibroblasts under serum induced condition (P > 1

Normalization	Parameters	Binding Motif	P-value	Correlation
MAS 5 (unfiltered)	Length 7 (0 IUPAC)	***CCACACA ***	2.44	-0.0376
		***GGGGCTC***	2.19	-0.0368
	
	Length 7 (1 IUPAC)	***CCACACA***	2.14	-0.0376
		***GGVCTC***	1.91	-0.0386

MAS 5 (filtered)	Length 7 (0 IUPAC)	***GGGGCTC***	2.28	-0.0500
	
	Length 7 (1 IUPAC)	***GGGGHTC***	2.56	-0.0573

RMA (filtered)	Length 7 (0 IUPAC)	GCGCCA	2.52	-0.0432
		***GTCCCG***	1.46	-0.0388
	
	Length 7 (1 IUPAC)	***GTCVCG***	4.29	-0.0545

Note: P-values are shown as -log_10 _values.

IUPAC characters R = A/G; W = T/A; H = A/T/C, V = A/C/G

**Table 4:** Possible gene targets and TFs found from the TRANSFAC database for top binding motifs from Table 3

Binding Motif	Possible gene targets	Possible TFs
***CCACACA***	-	-

***GGGGCTC***	ADA (adenosine deaminase)	SP1

***GTCCCG***	EGFR (EGF receptor)	-
	ATF2 (activating transcription factor 2)	SP1
	CCNE1 (cyclin E1)	E2F-1
	MET (hepatocyte growth factor receptor)	PAX-3

### Binding motifs found from fREDUCE for sets C and D suggest consistent effects from steroid induction for both keloid and normal fibroblasts

Binding motifs were found for set C (keloid treated with steroid versus serum induced keloid fibroblasts) and D (normal treated with steroid versus serum induced normal fibroblasts) when fREDUCE was run using parameters length 6 with 0 IUPAC substitutions. Other parameters did not produce any results. Furthermore, results were only obtained when MAS 5 normalization was used. The effect of hydrocortisone appears to be realized through the binding motifs GGAGGG and GCCCCC and this was consistent for both keloid (Table 5) and normal (Table 6) fibroblasts. A search through the TRANSFAC database using these binding motifs found a large list of genes containing these binding motifs, including COL1A2, FN, TGFB1, PDGF1 and IGF2 (Table 7). Of particular note is the fact that most of the genes found in this list have SP1 as its transcription factor (Table 7).

**Table 5:** Binding motifs found from fREDUCE for steroid treated versus control keloid fibroblasts (P > 1

Normalization	Parameters	Binding Motif	P-value	Correlation
MAS 5 (filtered)	Length 6 (0 IUPAC)	***GGAGGG***	24.62	-0.108
		***GCCCCC***	11	-0.0765
		CCTGGG	7.33	-0.0654
		TGTGTG	3.93	-0.0531
		GGCTGG	3.45	-0.0511
		CTGTGC	1.73	-0.0434
	
	Length 6 (1 IUPAC)	***GGWGGG***	30.68	-0.122
		CCDGGG	12.92	-0.0856
		CTCCCH	6.23	-0.0666
		TGTGDG	4.52	-0.0609
		HACGAA	3.63	0.0577
		ACCGCD	2.03	0.0514

Note: P-values are shown as -log_10 _values.

IUPAC characters W = T/A; H = A/T/C; V = A/C/G; D = A/T/G

**Table 6:** Binding motifs found from fREDUCE for steroid treated versus control normal fibroblasts (P > 1

Normalization	Parameters	Binding Motif	P-value	Correlation
MAS 5 (filtered)	Length 6 (0 IUPAC)	***GCCCCC***	30.08	-0.119
		***GGAGGG***	19.87	-0.0984
		CTGGGG	10.31	-0.0745
		TGGGCC	5.00	-0.0573
		CCCAGA	2.67	-0.0478
		AGAACG	2.44	0.0468
		TGGGTG	2.25	-0.0459
		GCGAAA	1.53	0.0425

Note: P-values are shown as -log_10 _values.

**Table 7:** Possible gene targets and TFs found from the TRANSFAC database for top binding motifs from Tables 5 and 6

Binding Motif	Possible gene targets	Possible TFs
***GGAGGG***	EPO (erythropoietin)	Tf-LF1 and Tf-LF2
	ATF2 (activating transcription factor 2)	SP1
	RARG (retinoic acid receptor, gamma)	SP1
	ACTC1 (actin, alpha, cardiac muscle 1)	SP1
	FN (fibronectin)	-
	c-myc	Pur factor
	CEACAM5 (carcinoembryonic antigen-related cell adhesion molecule 5)	SP1
	CYP17 (cytochrome P450, subfamily XVII)	PBX1B
	SFTPB (surfactant protein B)	NKX2-1
	PDGFA (platelet derived growth factor A chain)	SP1, WT1
	ADA (adenosine deaminase)	SP1
	SA-ACT (skeletal alpha actin)	COUP-TF2
	MIP (major intrinsic protein of lens fiber)	SP1
	c-myb	MZF-1
	COL1A2 (collagen I alpha 2)	SP1
	ALDC (aldolase C)	-

***GCCCCC***	TGFB1 (transforming growth factor beta 1)	SP1 and AP1
	apoE (apolipoprotein E)	-
	c-jun	SP1
	ACTC1 (actin, alpha, cardiac muscle 1)	SP1
	ATF2 (activating transcription factor 2)	SP1
	apoB (apolipoprotein B)	-
	GFAP (glial fibrillary acidic protein)	NF1, SP1
	Cyclin D1	c-Ets-2
	Insulin	-
	ALDC (aldolase C)	-
	HRAS (transforming protein p21)	SP1
	PFKM (muscle phosphofructokinase)	SP1
	DRD1 (dopamine receptor D1)	-
	IGF2 (insulin-like growth factor 2)	WT1

### Not many binding motifs found from fREDUCE for sets E and F

fREDUCE found few binding motifs for set E (keloid versus normal fibroblasts both treated with steroid) and no binding motifs for set F (keloid treated with HDGF versus untreated keloid fibroblasts). Binding motifs for set E were found only when the MAS 5 unfiltered condition and the RMA filtered condition were used (Table 8). Furthermore, binding motifs found in these conditions were not very consistent. A search through the TRANSFAC database using the top binding motifs from Table 8 found EGFR, ADM and CGA as possible gene targets (Table 9).

**Table 8:** Binding motifs found from fREDUCE for keloid versus normal fibroblasts under steroid treated condition (P > 1

Normalization	Parameters	Binding Motif	P-value	Correlation
MAS 5 (unfiltered)	Length 6 (0 IUPAC)	***CGCCGC ***	1.53	0.0314
	
	Length 6 (1 IUPAC)	GCGYTT	1.42	-0.0361

RMA (filtered)	Length 7 (0 IUPAC)	***GGGTTG***	2.75	0.0441
		CGTTTT	1.80	-0.0403
		AGCGAC	1.73	-0.0400

Note: P-values are shown as -log_10 _values

IUPAC character Y = C/T

**Table 9:** Possible gene targets and TFs found from the TRANSFAC database for top binding motifs from Table 8

Binding Motif	Possible gene targets	Possible TFs
***CGCCGC***	EGFR (EGF receptor)	SP1
	ADM (adrenomedullin)	TFAP2A

***GGGTTG***	CGA (glycoprotein hormone alpha subunit)	-

### Mean sensitivity performance of BANJO in recovering influence networks was significantly better than that of ARACNE

**Figure 2:**
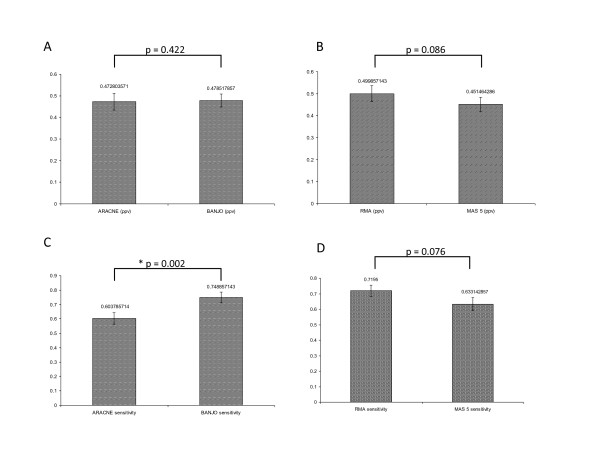
**Comparison between ARACNE, BANJO, RMA and MAS 5 based on PPV and sensitivity values**. (A) ARACNE (ppv) compared with BANJO (ppv). (B) RMA (ppv) compared with MAS 5 (ppv) (C) ARACNE (sensitivity) compared with BANJO (sensitivity) (D) RMA (sensitivity) compared with MAS 5 (sensitivity). Bar graphs represent mean ± S.E.M values. * indicates statistical significance as assessed by the paired t-test.

On average, BANJO was significantly more sensitive compared to ARACNE in recovering influence networks (Figure 2C). However, there was no significant difference in average accuracy (PPV) between BANJO and ARACNE (Figure 2A). Furthermore, there was no significant difference between RMA and MAS 5 normalization both in terms of mean accuracy (PPV) (Figure 2B) as well as mean sensitivity (Figure 2D) although p-values were fairly close to 0.05, with RMA being the better choice for both measures.

### Transcriptional networks were better suited for network inference compared to cytokine receptor interactions and intracellular signaling networks

Transcriptional networks (networks from Figure 1C, E and 1G) were better suited for network inference compared to cytokine receptor interactions (networks from Figure 1A and 1B) and intracellular signaling networks (networks from Figure 1D and 1F). The full list of results for all data sets is given in Table 10 with bold typeface indicating performance better than random. In particular, RMA normalization for transcriptional networks (C, E and G) had consistently better accuracy (PPV) compared to random, and also had sensitivity values higher than 0.5, regardless of the algorithm used. BANJO appears to perform particularly well for intracellular signaling network F, but did not do so well for network D. For the NFKB transcriptional network (network E), performance using keloid sets were consistently lower than performance using normal sets, but there was very little difference in performance between the keloid and normal datasets for the other networks (Table 10).

**Table 10:** PPV and sensitivity results for all the different pathways (A - G) run using BANJO and ARACNE

	A				B			
	ARACNE		BANJO		ARACNE		BANJO	
	PPV	Se	PPV	Se	PPV	Se	PPV	Se
Keloid RMA	0.238	0.556	0.3	0.667	**0.6**	1	0.4	0.667
Normal RMA	0.1875	0.333	0.3125	0.556	0.5	0.667	0.4	0.667
Keloid MAS5	0.214	0.333	0.286	0.667	0.5	0.667	**0.75**	1
Normal MAS5	0.25	0.667	0.304	0.778	**0.75**	1	0.6	1

Random	0.389				0.5			

	**C**				**D**			
	**ARACNE**		**BANJO**		**ARACNE**		**BANJO**	
	**PPV**	**Se**	**PPV**	**Se**	**PPV**	**Se**	**PPV**	**Se**

Keloid RMA	**0.8**	1	**0.75**	0.75	0.5	0.6	0.5	0.8
Normal RMA	**1**	0.75	**0.8**	1	0.5	0.6	0.2	0.2
Keloid MAS5	0.5	0.25	0.6	0.75	0.5	0.6	**0.625**	1
Normal MAS5	0.667	0.5	0.5	0.5	0.333	0.4	0.5	0.8

Random	0.667				0.5			

	**E**				**F**			
	**ARACNE**		**BANJO**		**ARACNE**		**BANJO**	
	**PPV**	**Se**	**PPV**	**Se**	**PPV**	**Se**	**PPV**	**Se**

Keloid RMA	**0.375**	0.6	**0.364**	0.8	0.375	0.75	**0.5**	1
Normal RMA	**0.444**	0.8	**0.444**	0.8	**0.5**	0.75	**0.5**	1
Keloid MAS5	0.286	0.4	0.273	0.6	0.286	0.5	**0.429**	0.75
Normal MAS5	0.333	0.6	**0.364**	0.8	0.2	0.25	**0.429**	0.75

Random	0.333				0.4			

	**G**							
	**ARACNE**		**BANJO**					
	**PPV**	**Se**	**PPV**	**Se**				
				
Keloid RMA	0.5	0.5	**0.714**	0.833				
Normal RMA	**0.667**	0.667	**0.625**	0.833				
Keloid MAS5	0.4	0.333	0.429	0.5				
Normal MAS5	**0.833**	0.833	0.5	0.5				
				
Random	0.6							

## Discussion

Reverse engineering gene networks from expression data is a considerably difficult problem, with challenges arising from the nature of the data which is typically noisy, high dimensional, and significantly undersampled. Most evaluations of reverse engineering techniques are done on simulated data [11,20] although some have extended this to small sets of experimental data [19,21]. While simulated data can model the high dimensionality as well as the indeterminacy of the problem accurately, the nature of noise as well as the underlying function governing the regulatory interactions has to be assumed *a priori*. A major problem of working with experimental data, however, is that not enough is known about the real networks and this could lead to difficulties in validating the inferred networks.

Our results from the physical approach show that MAS 5 normalization was better suited for the recovery of significant binding motifs as more binding motifs were obtained when the fREDUCE method was used. However, the results from influence methods show that RMA is better for the inference of gene networks especially for the case of transcriptional networks. The performance of different normalization approaches have been assessed in a previous study by Lim et al [22]. In the study, the Spearman rank correlation was used to compare between gene expression profile pairs from replicate samples as well as from samples with randomly permuted probe values. The authors found that the GCRMA procedure produced significant correlation artefacts (false positives), and that the MAS 5 procedure was best suited for the reverse engineering process. However, for most of their tests, RMA performs similarly, albeit at a lower level, compared to MAS 5. Here, we report that the performance of RMA or MAS 5 normalization appears to be dependent on the type of inference done.

The physical approach using the fREDUCE algorithm found binding motifs that were active in keloid fibroblasts compared to normal fibroblasts under various conditions. fREDUCE also found binding motifs that were responsive to steroid treatment. One limitation of the fREDUCE algorithm is that it cannot determine which TFs bind to the discovered motifs. By manually searching through the TRANSFAC database, we are able to get some idea about target genes containing these motifs, as well as possible transcription factors that bind to these motifs. The TRANSFAC database is not complete however, as it is based on published data, therefore undiscovered interactions will not be reflected in the database.

Our results suggest that steroid treatment affected both keloid and normal fibroblasts in a similar fashion as the top binding motifs found when these two cell types were treated with hydrocortisone were the same. Many of the possible gene targets containing these binding motifs are involved in wound healing, for example fibronectin, erythropoietin, PDGF, COL1A1 and TGFB. This is consistent with the fact that steroids are known to have a depressive effect on wound healing [23]. Furthermore, SP1 was the most common transcription factor found for these gene targets. This result suggests that hydrocortisone exerts its depressive effect on fibroblasts by affecting the activity of SP1, and could be a future area of research. There were fewer gene targets found for binding motifs that were active when comparing keloid to normal fibroblasts. Furthermore, the transcription factors found for these conditions were also less consistent. This could be due to the fact that the keloid condition is a result of the effect of multiple transcription factors, and unlike the effect of hydrocortisone, no single transcription factor is most responsible.

The success of fREDUCE depends on a number of assumptions regarding the dynamics of transcription. Most notably, it relates the influence of combinations of TFs as a log-linear function of RNA levels. Such a highly constrained model may lead to errors in predictions. Furthermore, it assumes that the 1000 base pairs upstream of the transcription start site play some role in the regulation of the gene. Despite these limitations, fREDUCE has been used successfully to discover binding motifs in human liver tissue [12]. We also used fairly naïve methods in the preprocessing of data for our influence based inference methods. To enable comparison between multiple datasets, we normalized the expression values with the average of GAPDH and B-actin expression values for each individual chip whereas for discretization, we used the 7 bin quantile discretization that is available in BANJO. More sophisticated discretization techniques [24-26] might potentially produce better results.

Due to our limited data, it would be unwise to run the influence algorithms on the full list of genes. The subsets of genes that we selected were based on KEGG pathways that were found to be significantly enriched in our previous study. We further subdivided the pathways found into three major groups - cytokine receptor interactions, transcriptional networks and intracellular signaling. These lists are by no means complete, and there is bound to be many hidden factors and feedback interactions that were not explicitly modeled or taken into account. However, in the absence of further biological knowledge to guide us in our selection, this seemed to be the most logical step to take. Furthermore, it is hoped that the influence methods, being of the 'black box' variety, would be able to cope with these deficiencies.

Our results show that both ARACNE and BANJO seem to perform better for transcriptional networks compared to cytokine receptor interations or intracellular signaling networks. This makes some intuitive sense as there is a causal link between transcription factors and their target genes, whereas in cytokine receptor interactions and in intracellular signaling, there is at most only a correlation (in some cases, there may not even be a correlation). However, a cellular signaling network has been successfully reconstructed previously using a Bayesian approach [9]. It should be noted however that Sach's study measured phosphorylation levels in addition to expression levels and used the flow cytometry platform instead of microarray expression data. On a related note, it is worth pointing out that influence methods using microarray data do not take the actual binding of transcription factors into consideration as only expression values are used. This could be a source of inaccuracies in the networks inferred. Therefore, improved methods that model alternate regulatory mechanisms such as post translational modifications and binding affinities could be developed to improve the accuracy of the reverse engineering process.

Between the two influence methods tested, BANJO produced significantly better results compared to ARACNE. The superior performance of BANJO for small data sets with 'global' perturbations can also be seen in the results of the *in silico *study done by Bansal et al. [19]. The lower performance of ARACNE could be due to the small number of data sets used; ARACNE has been recommended to be used on data sets containing a minimum of 100 microarray expression profiles as this represents an empirical lower bound on the amount of data needed to estimate the mutual information reliably [27]. Having said that, none of the PPV values obtained either through BANJO or ARACNE was able to beat the random score significantly as assessed by the chi-squared test, although the absolute PPV values were higher in some of the cases. Achieving statistical significance with a small number of genes requires the difference in data distributions to be very large and may be too demanding for our small networks.

The fact that performance for the transcriptional network involving NFKB (nuclear factor kappa-light-chain-enhancer of activated B cells, Network E) using influence methods was better for normal fibroblasts compared to keloid fibroblasts suggests that the influence between NFKB and its targets was weaker in keloid fibroblasts, or that there were more links in the keloid network that were not captured by our simplified diagram. This would imply that targeting of NFKB alone may not be sufficient in reducing the expression of its targets in keloids. However, more work needs to be done to verify this hypothesis.

## Conclusions

In this study, we have attempted to reverse engineer gene networks from all microarray expression data that we have collected. Using the physical approach of correlating expression values to binding motifs, we found some consensus sequences that were active in the keloid condition, as well as some sequences that were responsive to steroid treatment which is one of the commonly used treatments for keloids. These consensus sequences are possible transcription factor binding sites and could be explored for developing future keloid treatments or to improve the efficacy of current steroid treatments. Using influence approaches on experimental data, we found that the combination of the Bayesian algorithm, RMA normalization and transcriptional networks gave the best reconstruction results and this could serve as a guide for future influence approaches dealing with experimental data. Furthermore, our results show that the NFKB transcriptional network inferred from normal fibroblast data was more accurate than that inferred from keloid data, suggesting a more robust network in the keloid condition.

The ability to infer molecular interactions in cellular systems is one of the most exciting promises of systems biology. As the most widely available high throughput technology, gene expression microarrays provide a good test set for the application of inference algorithms that infer dynamic models from static, genome-scale data. However, the critical assumption underlying this methodology is that mRNA measurements are predictive of molecular activity. This assumption has been thrown into question as new studies reveal the substantial role of alternate regulatory mechanisms, such as translation, post-translational modifications, genetic and epigenetic factors, as well as the increasingly appreciated regulatory role of non-coding RNAs. Furthermore, data from the microarray platform is typically noisy, and is also hidden in multiple probes that can be combined in multiple ways to produce different expression values. Yet in spite of all these difficulties, the topic of reverse engineering gene networks is surely worth pursuing, as it provides us with a means of understanding biology not only in terms of the genes themselves, but also through their interactions.

## Competing interests

The authors declare that they have no competing interests.

## Authors' contributions

BNSO conducted the experiments, carried out computational analysis and drafted the manuscript. PTT made contributions in the design of the experiments, interpretation of biological data and in the drafting of the manuscript. All authors have read and approved the final manuscript.
